# Supplementary material for: Quantifying infectious disease epidemic risks: A practical approach for seasonal pathogens
Source: PLoS Comput Biol. 2025 Feb 19;21(2):e1012364. doi: 10.1371/journal.pcbi.1012364 (PMC11867399; doi:10.1371/journal.pcbi.1012364)
Supplement: S1 Text — (DOCX) [file pcbi.1012364.s001.docx]

**Supplementary text for *Quantifying infectious disease epidemic risks: A practical approach for seasonal pathogens***

**Section A. Parameters of the chikungunya transmission model**

The parameters of the model of chikungunya virus transmission are listed in Table A, along with the assumed parameter values. For the temperature-dependent parameter values, a functional form is provided (based on the temperature, $T$).

**Table A. Parameters of the *Ae. albopictus* population model and the chikungunya transmission model (systems of equations (3) and (6) in the main text).** Graphical representations of parameters that are temperature-dependent are shown in Poletti *et al.* [1]. Relevant references from which the assumed values and functional forms were obtained are shown in the final column.

| **Parameter** | **Interpretation** | **Value** | **Units** | **Reference** |
| --- | --- | --- | --- | --- |
| $n_{E}$ | Average number of eggs per adult female oviposition | 60 | - | [1–3] |
| $d_{E}(T)$ | Development rate from egg to larva | $\frac{1}{6.9-4e^{-\left( \frac{T-20}{4.1} \right)^{2}}}$ | $\text{days}^{-1}$ | [1–3] |
| $d_{L}(T)$ | Development rate from larva to pupa | $\frac{1}{0.12T^{2}-6.6T+98}$ | $\text{days}^{-1}$ | [1–3] |
| $d_{P}(T)$ | Development rate from pupa to adult | $\frac{1}{0.027T^{2}-1.7T+27.7}$ | $\text{days}^{-1}$ | [1–3] |
| $d_{V}(T)$ | Rate of egg deposition for female adults | $\frac{1}{0.046T^{2}-2.77T+45.3}$ | $\text{days}^{-1}$ | [1–3] |
| $m_{E}(T)$ | Egg mortality rate | $506-506e^{-\left( \frac{T-25}{27.3} \right)^{6}}$ | $\text{days}^{-1}$ | [1–3] |
| $m_{L}(T)$ | Larval mortality rate | $0.029+858e^{T-43.4}$ | $\text{days}^{-1}$ | [1–3] |
| $m_{P}(T)$ | Pupal mortality rate | $0.021+37e^{T-36.8}$ | $\text{days}^{-1}$ | [1–3] |
| $m_{V}(T)$ | Adult vector mortality rate | $0.031+95820e^{T-50.4}$ | $\text{days}^{-1}$ | [1–3] |
| $k$ | Adult vector biting rate | 0.09 | $\text{days}^{-1}$ | [1,2] |
| $\beta_{V}$ | Probability of vector infection from a single blood meal from an infectious host | 0.77 | - | [2] |
| $\beta_{H}$ | Probability of host infection from a single blood meal from an infectious vector | 0.70 | - | [2] |
| $\omega_{V}$ | Extrinsic incubation period of the vector | 2.5 | $\text{days}$ | [2] |
| $\tau$ | Time until host recovery | 4.5 | $\text{days}$ | [2] |
| $a_{s}$ | Overcrowding term | 44.5 (2014), 32.6 (2015) | - | [2] |

**Section B. Supplementary details about the algorithms used for outbreak simulations**

Algorithm A. Modified Gillespie algorithm applied to the SIR model

Two possible events can occur in the stochastic SIR model. These events, and the rates at which they occur, are shown in Table B.

**Table B. Possible events in the stochastic SIR model.**

| **Event** | **Rate** |
| --- | --- |
| A randomly chosen susceptible individual becomes infected | $\frac{\beta\left( t \right)S\left( t \right)I\left( t \right)}{N}$ |
| A randomly chosen infected individual is removed | $\gamma\left( t \right)I\left( t \right)$ |

A single realisation of the stochastic SIR model can be simulated using the following algorithm:

1. Set the initial time $t$ and the values of $S\left( t \right), I\left( t \right), R(t)$.
2. Steps 2-3 should be repeated while the outbreak is still ongoing (i.e. $I\left( t \right)>0$) or until the user chooses to stop the simulation. Calculate the time of the next event, $t+\Delta\tau$, using the expression

$$\int_{t}^{t+\Delta\tau} \frac{\beta\left( s \right)S\left( s \right)I\left( s \right)}{N}+\gamma\left( s \right)I\left( s \right)\text{d}s=-\ln\left( r_{1} \right),$$

where $r_{1}$ is a random number sampled from a uniform distribution on $(0,1)$.

1. Determine whether the next event is an infection event or a removal event. To do this, sample a second random number ($r_{2}$) from a uniform distribution on $(0,1)$. If

$$r_{2}<\frac{\beta\left( t+\Delta\tau\right)S\left( t \right)I\left( t \right)/N}{\beta\left( t+\Delta\tau\right)S\left( t \right)I\left( t \right)/N+\gamma(t+\Delta\tau)I(t)},$$

then the next event is an infection event; set $S\left( t+\Delta\tau\right)=S\left( t \right)-1$, $I\left( t+\Delta\tau\right)=I\left( t \right)+1$, and $R\left( t+\Delta\tau\right)=R\left( t \right).$ If instead the inequality above is not satisfied, then the next event is a removal event; set $S\left( t+\Delta\tau\right)=S\left( t \right)$, $I\left( t+\Delta\tau\right)=I\left( t \right)-1$, and $R\left( t+\Delta\tau\right)=R\left( t \right)+1.$ Update the current time, $t$ (i.e. set $t$ to be $t+\Delta\tau$).

Algorithm B. Modified Gillespie algorithm applied to the chikungunya transmission model

As described in the main text, to simulate the chikungunya transmission model, we begin by solving the deterministic ecological model (system of equations (3) in the main text) numerically.Following the approach of Guzzetta *et al.* [2,4], the ecological model is initialised on 1^st^ April of the respective years with 10,000 eggs and no individuals in all other classes. We then fit equation (5) in the main text to the output from system of equations (3) in the main text to obtain a time series describing the number of adult female vectors each day, $N_{V}\left( t \right)$.

We then proceed by simulating the epidemiological component of the system (the analogous stochastic model to system of equations (6) in the main text). Within each day, transmission parameter values are assumed to remain constant. Seven possible events can occur in the simulations within each day (Table C).

**Table C. Possible events within each day in the stochastic chikungunya transmission model.**

| **Event** | **Rate** |
| --- | --- |
| A randomly chosen susceptible vector dies | $m_{V}(T(t))S_{V}$ |
| A randomly chosen susceptible vector gets infected and becomes exposed | $k\beta_{V}\frac{S_{V}I_{H}}{N}$ |
| A randomly chosen exposed vector dies | $m_{V}\left( T(t) \right)E_{V}$ |
| A randomly chosen exposed vector becomes infectious | $\frac{1}{\omega_{V}}E_{V}$ |
| A randomly chosen infectious vector dies | $m_{V}\left( T(t) \right)I_{V}$ |
| A randomly chosen susceptible host becomes infectious | $k\beta_{H}\frac{S_{H}I_{V}}{N}$ |
| A randomly chosen infectious host is removed | $\frac{1}{\tau}I_{H}$ |

The stochastic host-vector model is then simulated using the following steps:

1. Set the initial time $t$, and the values of $S_{V}(t)$, $E_{V}(t)$, $I_{V}(t)$, $S_{H}(t)$, $I_{H}(t)$, and $R_{H}\left( t \right)$. $S_{V}(t)$ is set to be $N_{V}(t)$ (from the deterministic ecological model), $S_{H}(t)$ is set to be the number of people living within the simulation area, and $I_{H}(t)$ is set to be one (this is the initial infection). All other compartments are initialised with zero individuals.
2. Steps 2-3 should be repeated while the outbreak is still ongoing (i.e. $E_{V}+I_{V}+I_{H}>0$) or until the user chooses to stop the simulation. Propose the next event time, $t+\Delta\tau$, using the expression

$$\Delta\tau=-\frac{\ln r_{1}}{\theta\left( t \right)},$$

where $\theta\left( t \right)=m_{V}\left( T\left( t \right) \right)S_{V}(t)+k\beta_{V}\frac{S_{V}(t)I_{H}\left( t \right)}{N}+m_{V}\left( T(t) \right)E_{V}(t)+\frac{1}{\omega_{V}}E_{V}(t)+m_{V}\left( T(t) \right)I_{V}(t)+k\beta_{H}\frac{S_{H}\left( t \right)I_{V}\left( t \right)}{N}+\frac{1}{\tau}I_{H}(t)$ and $r_{1}$ is a random number sampled from a uniform distribution on $(0,1)$.

1. Then, do one of the following, depending on the proposed next event time ($t+\Delta\tau$):

• If this proposed time $t+\Delta\tau$ is not in the same day as time $t$ (i.e. if $\left\lfloor t+\Delta\tau\right\rfloor-\left\lfloor t \right\rfloor>0$), then do not perform any event and instead update the time to be the end of the original day (i.e. update $t$ to $\left\lceil t \right\rceil$). Update the vector population size according to the deterministic ecological model (i.e., $N_{V}\left( t \right)$). Let $\sigma\left( t \right)=N_{V}\left( t \right)-S_{V}\left( t \right)-E_{V}\left( t \right)-I_{V}\left( t \right)$. If $\sigma\left( t \right)>0$, then add $\sigma\left( t \right)$ individuals to $S_{V}\left( t \right)$. If $\sigma\left( t \right)<0$, then remove $-\sigma\left( t \right)$ individuals from $S_{V}\left( t \right)$, $E_{V}\left( t \right)$ or $I_{V}\left( t \right)$ (with each vector to remove chosen uniformly at random from those compartments).

• If instead the proposed time $t+\Delta\tau$ is in the same day as $t$ (i.e. if $\left\lfloor t+\Delta\tau\right\rfloor-\left\lfloor t \right\rfloor=0$), do not update the vector population size. Determine the type of the event occurring at time $t+\Delta\tau$ (since transmission parameters are assumed to be constant within each day, this is equivalent to deploying the Gillespie direct method within each day). To do this, define $\theta_{i}(t)$ to be the sum of the first $i$ terms of $\theta\left( t \right)$, so that $\theta_{1}\left( t \right)=m_{V}\left( T\left( t \right) \right)S_{V}(t)$, $\theta_{2}\left( t \right)=m_{V}\left( T(t) \right)S_{V}(t)+k\beta_{V}\frac{S_{V}(t)I_{H}\left( t \right)}{N}$, and so on. Then, sample a second number ($r_{2}$) from a uniform distribution on $(0,1)$, and perform one of the following events.

- If $r_{2}<\frac{\theta_{1}\left( t \right)}{\theta\left( t \right)}$, then the next event is the death of a susceptible vector; set $S_{V}\left( t+\Delta\tau\right)=S_{V}\left( t \right)-1$, $E_{V}\left( t+\Delta\tau\right)=E_{V}(t)$, $I_{V}\left( t+\Delta\tau\right)=I_{V}(t)$, $S_{H}\left( t+\Delta\tau\right)=S_{H}(t)$, $I_{H}\left( t+\Delta\tau\right)=I_{H}(t)$, and $R_{H}\left( t+\Delta\tau\right)=R_{H}\left( t \right)$.

- If instead, ${\frac{\theta_{1}\left( t \right)}{\theta\left( t \right)}<r}_{2}<\frac{\theta_{2}\left( t \right)}{\theta\left( t \right)}$, then the next event is a susceptible vector getting infected and becoming exposed; $S_{V}\left( t+\Delta\tau\right)=S_{V}\left( t \right)-1$, $E_{V}\left( t+\Delta\tau\right)=E_{V}\left( t \right)+1$, $I_{V}\left( t+\Delta\tau\right)=I_{V}(t)$, $S_{H}\left( t+\Delta\tau\right)=S_{H}(t)$, $I_{H}\left( t+\Delta\tau\right)=I_{H}(t)$, and $R_{H}\left( t+\Delta\tau\right)=R_{H}\left( t \right)$.

- If instead, ${\frac{\theta_{2}\left( t \right)}{\theta\left( t \right)}<r}_{2}<\frac{\theta_{3}\left( t \right)}{\theta\left( t \right)}$, then the next event is the death of an exposed vector; $S_{V}\left( t+\Delta\tau\right)=S_{V}(t)$, $E_{V}\left( t+\Delta\tau\right)=E_{V}\left( t \right)-1$, $I_{V}\left( t+\Delta\tau\right)=I_{V}(t)$, $S_{H}\left( t+\Delta\tau\right)=S_{H}(t)$, $I_{H}\left( t+\Delta\tau\right)=I_{H}(t)$, and $R_{H}\left( t+\Delta\tau\right)=R_{H}\left( t \right)$.

- If instead, ${\frac{\theta_{3}\left( t \right)}{\theta\left( t \right)}<r}_{2}<\frac{\theta_{4}\left( t \right)}{\theta\left( t \right)}$, then the next event is the transition of an exposed vector into an infectious vector; set$S_{V}\left( t+\Delta\tau\right)=S_{V}(t)$, $E_{V}\left( t+\Delta\tau\right)=E_{V}\left( t \right)-1$, $I_{V}\left( t+\Delta\tau\right)=I_{V}\left( t \right)+1$, $S_{H}\left( t+\Delta\tau\right)=S_{H}(t)$, $I_{H}\left( t+\Delta\tau\right)=I_{H}(t)$, and $R_{H}\left( t+\Delta\tau\right)=R_{H}\left( t \right)$.

- If instead, ${\frac{\theta_{4}\left( t \right)}{\theta\left( t \right)}<r}_{2}<\frac{\theta_{5}\left( t \right)}{\theta\left( t \right)}$, then the next event is the death of an infectious vector; set $S_{V}\left( t+\Delta\tau\right)=S_{V}(t)$, $E_{V}\left( t+\Delta\tau\right)=E_{V}(t)$, $I_{V}\left( t+\Delta\tau\right)=I_{V}\left( t \right)-1$, $S_{H}\left( t+\Delta\tau\right)=S_{H}(t)$, $I_{H}\left( t+\Delta\tau\right)=I_{H}(t)$, and $R_{H}\left( t+\Delta\tau\right)=R_{H}\left( t \right)$.

- If instead, ${\frac{\theta_{5}\left( t \right)}{\theta\left( t \right)}<r}_{2}<\frac{\theta_{6}\left( t \right)}{\theta\left( t \right)}$, then the next event is the infection of a susceptible host; set $S_{V}\left( t+\Delta\tau\right)=S_{V}(t)$, $E_{V}\left( t+\Delta\tau\right)=E_{V}(t)$, $I_{V}\left( t+\Delta\tau\right)=I_{V}(t)$, $S_{H}\left( t+\Delta\tau\right)=S_{H}\left( t \right)-1$, $I_{H}\left( t+\Delta\tau\right)=I_{H}\left( t \right)+1$, and $R_{H}\left( t+\Delta\tau\right)=R_{H}\left( t \right)$.

- If instead, ${\frac{\theta_{6}\left( t \right)}{\theta\left( t \right)}<r}_{2}<1$, then the next event is the removal of an infectious host; set $S_{V}\left( t+\Delta\tau\right)=S_{V}(t)$, $E_{V}\left( t+\Delta\tau\right)=E_{V}(t)$, $I_{V}\left( t+\Delta\tau\right)=I_{V}(t)$, $S_{H}\left( t+\Delta\tau\right)=S_{H}(t)$, $I_{H}\left( t+\Delta\tau\right)=I_{H}\left( t \right)-1$, and $R_{H}\left( t+\Delta\tau\right)=R_{H}\left( t \right)+1$.

Then update the current time, $t$ (i.e. set $t$ to be $t+\Delta\tau$).

**Section C. Derivation of the CER for the host-vector model**

We derive the CER for the host-vector model, considering a scenario in which a single infectious host enters the population at time $t=t_{0}$. To do this, we denote the probability of a major epidemic failing to develop starting from $i$ infectious hosts, $j$ exposed vectors and $k$ infectious vectors in the population at time $t=t_{0}$ by $q_{ijk}(t_{0})$.

We begin by assuming that there is one infectious host and no exposed or infectious vectors in the population in order to write down an equation for the temporal evolution of $q_{100}$. We then consider the possible events in the next $\Delta t$ months (i.e. the time interval $[t_{0},t_{0}+\Delta t]$), where $\Delta t$ represents a very short time period so that at most a single event is possible. In that time period, the probability that a vector gets infected (i.e. transitions from the susceptible compartment to the exposed compartment) is approximately $k\beta_{V}\frac{S_{V}I_{H}}{N}\Delta t$; the probability that the infectious host recovers is approximately $\frac{1}{\tau}\Delta t$; and the probability that no event occurs is approximately $1-k\beta_{V}\frac{S_{V}I_{H}}{N}\Delta t-\frac{1}{\tau}\Delta t$. Applying the law of total probability therefore gives

$$q_{100}\left( t_{0} \right) =\text{Prob}\left( \text{vector infection event occurs in} \left[ t_{0},t_{0}+\Delta t \right] \right)\times\text{Prob}\left( \text{no major outbreak} \right| \text{vector infection event occurs in} \left[ t_{0},t_{0}+\Delta t \right])+\text{Prob}\left( \text{host recovery event occurs in} \left[ t_{0},t_{0}+\Delta t \right] \right)\times\text{Prob}\left( \text{no major outbreak} \right| \text{host recovery event occurs in} \left[ t_{0},t_{0}+\Delta t \right])+\text{Prob}\left( \text{no event occurs in} \left[ t_{0},t_{0}+\Delta t \right] \right)\times\text{Prob}\left( \text{no major outbreak} \right| \text{no event occurs in} \left[ t_{0},t_{0}+\Delta t \right])$$

$$=k\beta_{V}\frac{S_{V}I_{H}}{N}\Delta tq_{110}\left( t_{0}+\Delta t \right)+\frac{1}{\tau}\Delta tq_{000}\left( t_{0}+\Delta t \right)+\left( 1-k\beta_{V}\frac{S_{V}I_{H}}{N}\Delta t-\frac{1}{\tau}\Delta t \right)q_{100}\left( t_{0}+\Delta t \right).$$

We make the assumption that infection lineages are independent (so that $q_{110}\left( t_{0}+\Delta t \right)=q_{100}\left( t_{0}+\Delta t \right)q_{010}\left( t_{0}+\Delta t \right)$). We note that $q_{000}\left( t_{0}+\Delta t \right)=1$, and that $S_{V}=N_{V}$ and $I_{H}=1$ at the beginning of the outbreak. Then, rearranging the expression above and taking the limit $\Delta t\to0$ gives

$$\frac{\text{d}q_{100}(t_{0})}{\text{d}t_{0}}=-k\beta_{V}\frac{N_{V}}{N}q_{100}\left( t_{0} \right)q_{010}\left( t_{0} \right)-\frac{1}{\tau}+\left( k\beta_{V}\frac{N_{V}}{N}+\frac{1}{\tau} \right)q_{100}\left( t_{0} \right).$$

Denoting the probability of a major outbreak starting from $i$ infectious hosts, $j$ exposed vectors and $k$ infectious vectors in the population at time $t=t_{0}$ by $p_{ijk}(t_{0})=1-q_{ijk}(t_{0})$ and substituting this into the above equation gives the first equation in system of equations (9) in the main text. An analogous approach (this time for $q_{010}$ and $q_{001}$; i.e. starting from either a single exposed vector or from a single infectious vector) is used to derive the remaining two equations in system of equations (9) in the main text.

**References**

1. Poletti P, Messeri G, Ajelli M, Vallorani R, Rizzo C, Merler S. Transmission potential of chikungunya virus and control measures: The case of Italy. PLoS One. 2011;6. doi:10.1371/journal.pone.0018860

2. Guzzetta G, Trentini F, Poletti P, Baldacchino FA, Montarsi F, Capelli G, et al. Effectiveness and economic assessment of routine larviciding for prevention of chikungunya and dengue in temperate urban settings in Europe. PLoS Negl Trop Dis. 2017;11. doi:10.1371/journal.pntd.0005918

3. Delatte H, Gimonneau G, Triboire A, Fontenille D. Influence of temperature on immature development, survival, longevity, fecundity, and gonotrophic cycles of *Aedes albopictus*, vector of chikungunya and dengue in the Indian Ocean. J Med Entomol. 2009;46: 33–41. doi:10.1603/033.046.0105

4. Guzzetta G, Montarsi F, Baldacchino FA, Metz M, Capelli G, Rizzoli A, et al. Potential risk of dengue and chikungunya outbreaks in Northern Italy based on a population model of *Aedes albopictus* (Diptera: Culicidae). PLoS Negl Trop Dis. 2016;10. doi:10.1371/journal.pntd.0004762
