# Supplementary material for: Quantifying infectious disease epidemic risks: A practical approach for seasonal pathogens
Source: PLoS Comput Biol. 2025 Feb 19;21(2):e1012364. doi: 10.1371/journal.pcbi.1012364 (PMC11867399; doi:10.1371/journal.pcbi.1012364)
Supplement: S1 Fig — A. Daily mean temperature in Feltre in 2014 as sourced from MODIS satellite Land Surface Temperature measurements (blue line) and smoothed temperature values obtained by fitting equation (4) in the main text to those data (orange line). B. Analogous to panel A, but using temperature data from 2015. C. Monthly number of adult female vectors per hectare in 2014 (and early 2015) obtained by solving system of equations (3) in the main text numerically based on the fitted temperature values in panel A (blue dots), and inferred number of adult female vectors per hectare obtained by fitting equation (5) in the main text to the monthly values (blue line). The ecological model is initialised at the beginning of April 2014. The fitted values shown from January to March 2014 reflect the fit from early 2015 (assuming annual periodicity). D. Analogous to panel C, but for 2015 (and early 2016), based on the fitted temperature values in panel B. (PDF) [file pcbi.1012364.s002.pdf]

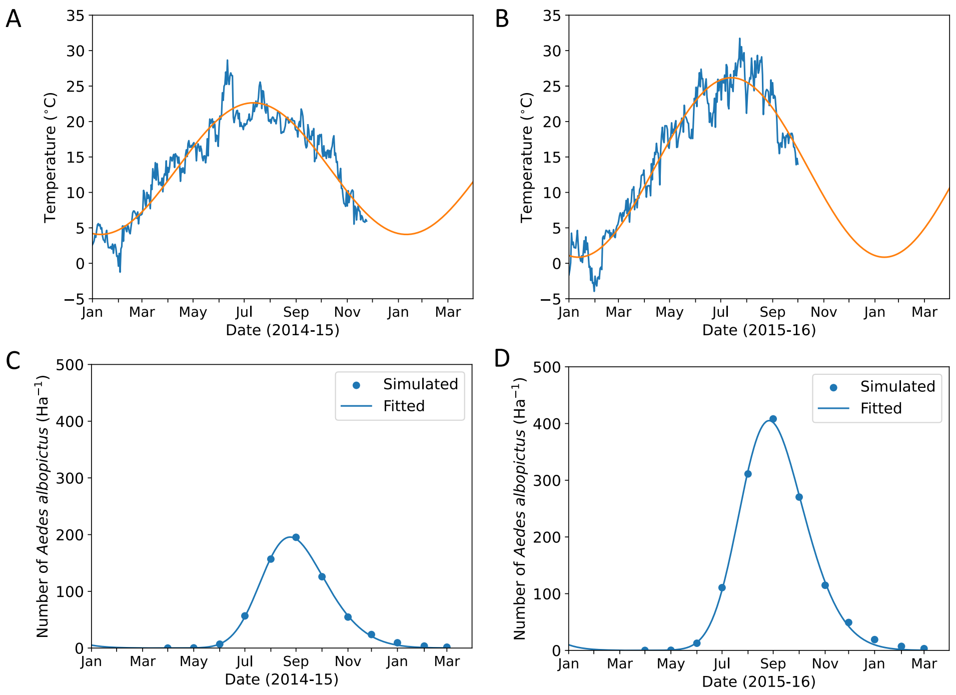

S1 Fig. Temperature and vector density in Feltre, Northern Italy, in 2014 (left column) and 2015 (right column). A. Daily mean temperature in Feltre in 2014 as sourced from MODIS satellite Land Surface Temperature measurements [1] (blue line) and smoothed temperature values obtained by fitting equation (4) in the main text to those data (orange line). B. Analogous to panel A, but using temperature data from 2015. C. Monthly number of adult female vectors per hectare in 2014 (and early 2015) obtained by solving system of equations (3) in the main text numerically based on the fitted temperature values in panel A (blue dots), and inferred number of adult female vectors per hectare obtained by fitting equation (5) in the main text to the monthly values (blue line). The ecological model is initialised at the beginning of April 2014, following the approach of Guzzetta *et al.* [1]. The fitted values shown from January to March 2014 reflect the fit from early 2015 (assuming annual periodicity). D. Analogous to panel C, but for 2015 (and early 2016), based on the fitted temperature values in panel B.

References

1. Guzzetta G, Montarsi F, Baldacchino FA, Metz M, Capelli G, Rizzoli A, et al. Potential risk of dengue and chikungunya outbreaks in Northern Italy based on a population model of *Aedes albopictus* (Diptera: Culicidae). PLoS Negl Trop Dis. 2016;10. doi:10.1371/journal.pntd.0004762
