# Supplementary material for: Quantifying infectious disease epidemic risks: A practical approach for seasonal pathogens
Source: PLoS Comput Biol. 2025 Feb 19;21(2):e1012364. doi: 10.1371/journal.pcbi.1012364 (PMC11867399; doi:10.1371/journal.pcbi.1012364)
Supplement: S2 Fig — A. The TER for different initial numbers of infectious individuals (obtained by solving system of equations (11) in the main text numerically). B. The duration of the year for which the TER exceeds z = 0 . 1, for different initial numbers of infectious individuals. In both panels, a threshold of M = 100 cumulative infections and a time step of Δt = 0.00033 months was used when computing the TER. The overall population size was assumed to be N = 1 , 000 individuals. Parameter values used: β0 = 4, and γ = 4 . 9 month-1. (PDF) [file pcbi.1012364.s003.pdf]

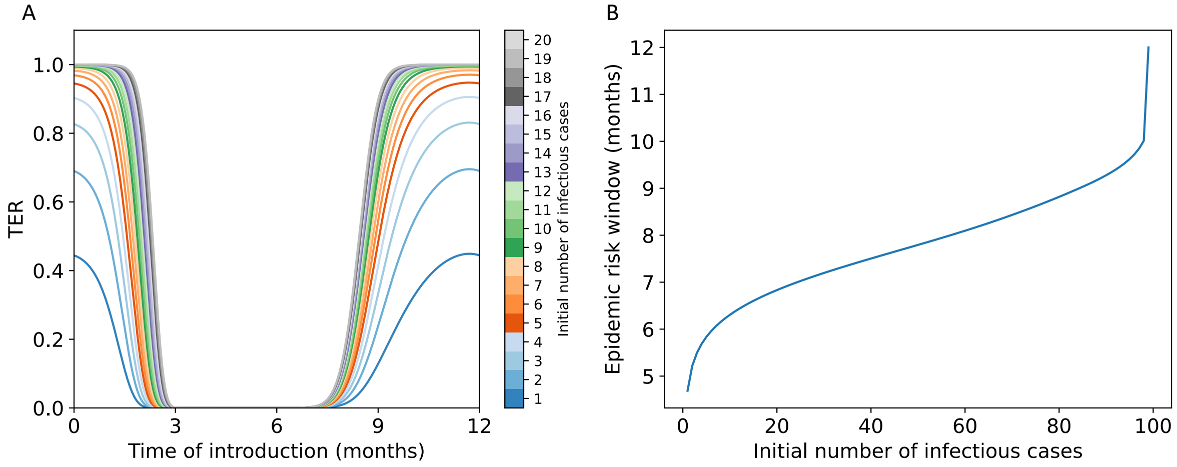

S2 Fig. Dependence of the TER on the initial number of infected individuals, for the stochastic SIR model with seasonal transmission. A. The TER for different initial numbers of infectious individuals (obtained by solving system of equations (11) in the main text numerically). B. The duration of the year for which the TER exceeds $z = 0.1$, for different initial numbers of infectious individuals. In both panels, a threshold of $M = 100$ cumulative infections and a time step of $\Delta t = 0.00033$ months was used when computing the TER. The overall population size was assumed to be $N = 1{,}000$ individuals. Parameter values used: $\beta_0 = 4$, $\beta_1 = 5$ and $\gamma = 4.9$ month^{-1}.
