# Supplementary material for: Quantifying infectious disease epidemic risks: A practical approach for seasonal pathogens
Source: PLoS Comput Biol. 2025 Feb 19;21(2):e1012364. doi: 10.1371/journal.pcbi.1012364 (PMC11867399; doi:10.1371/journal.pcbi.1012364)
Supplement: S3 Fig — A. The TER (obtained by solving system of equations (11) numerically) when sustained transmission is possible throughout the year (β0 = 10, and γ = 4 . 9 month-1), for different values of M (100, 200, 300, 400 and 500). For M = 500, the TER as approximated using model simulations is also plotted (blue dots). B. Analogous results to panel A, but in a scenario in which sustained transmission can only occur for some of the year (β0 = 4, β1 = 5 and γ = 4 . 9 month-1). In both panels, the overall population size was assumed to be N = 1 , 000 individuals. When we computed the TER numerically, we used the time step Δt = 0.00033 months. When we approximated the TER using model simulations, 10 , 000 simulations were run for each time of introduction considered. (PDF) [file pcbi.1012364.s004.pdf]

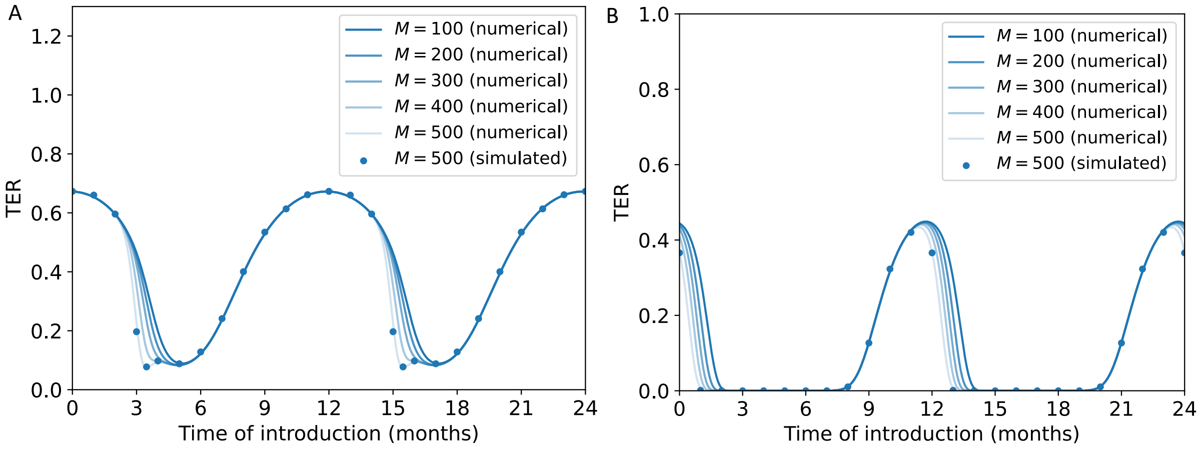

S3 Fig. Dependence of the TER on the value of M used, for the stochastic SIR model with seasonal transmission. A. The TER (obtained by solving system of equations (11) numerically) when sustained transmission is possible throughout the year ($\beta_0 = 10$, $\beta_1 = 5$ and $\gamma = 4.9$ month^{-1}), for different values of M (100, 200, 300, 400 and 500). For $M = 500$, the TER as approximated using model simulations is also plotted (blue dots). B. Analogous results to panel A, but in a scenario in which sustained transmission can only occur for some of the year ($\beta_0 = 4$, $\beta_1 = 5$ and $\gamma = 4.9$ month^{-1}). In both panels, the overall population size was assumed to be $N = 1{,}000$ individuals. When we computed the TER numerically, we used the time step $\Delta t = 0.00033$ months. When we approximated the TER using model simulations, $10{,}000$ simulations were run for each time of introduction considered.
