# Supplementary material for: Quantifying infectious disease epidemic risks: A practical approach for seasonal pathogens
Source: PLoS Comput Biol. 2025 Feb 19;21(2):e1012364. doi: 10.1371/journal.pcbi.1012364 (PMC11867399; doi:10.1371/journal.pcbi.1012364)
Supplement: S4 Fig — A. The CER (obtained using equation (8) in the main text; blue line) and the TER (obtained by solving system of equations (11) in the main text numerically; orange line) when β0 = 10, β1 = 0 and γ = 4 . 9 month-1. B. Analogous results to panel A, but with β1 = 3. C. Analogous results to panel A, but with β1 = 6. D. Analogous results to panel A, but with β1 = 9. In all panels, a threshold of M = 100 and a time step of Δt = 0.00033 months was used when computing the TER. The overall population size was assumed to be individuals. Insets show R0 ( t ) = β ( t ) / γ ( t ) as a function of t. (PDF) [file pcbi.1012364.s005.pdf]

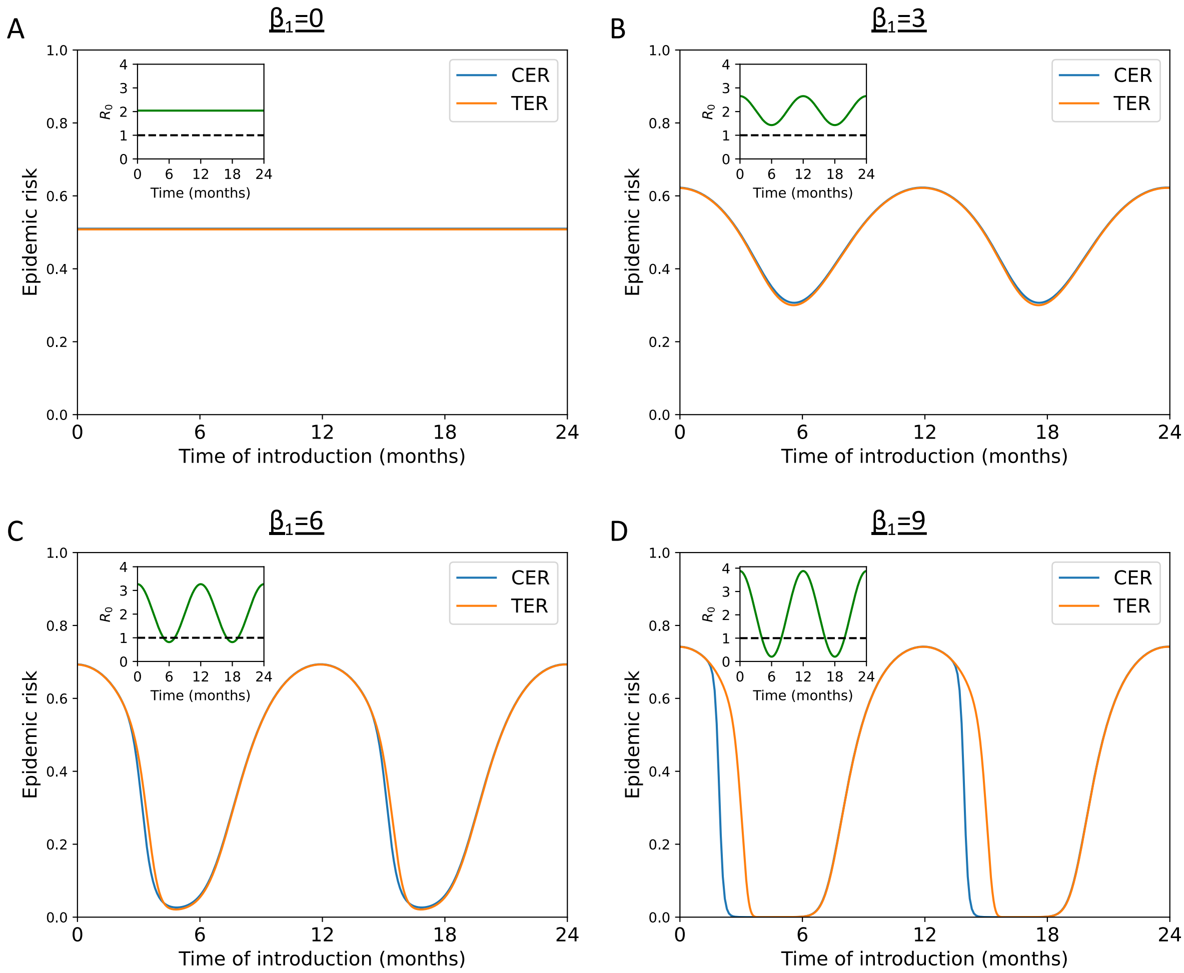

S4 Fig. Comparison between calculated values of the CER and TER for the stochastic SIR model with seasonal transmission, for a range of values of $\boldsymbol{\beta_1}$. A. The CER (obtained using equation (8) in the main text; blue line) and the TER (obtained by solving system of equations (11) in the main text numerically; orange line) when $\beta_0 = 10$, $\beta_1 = 0$ and $\gamma = 4.9$ month^{-1}. B. Analogous results to panel A, but with $\beta_1 = 3$. C. Analogous results to panel A, but with $\beta_1 = 6$. D. Analogous results to panel A, but with $\beta_1 = 9$. In all panels, a threshold of $M = 100$ and a time step of $\Delta t = 0.00033$ months was used when computing the TER. The overall population size was assumed to be $N = 1{,}000$ individuals. Insets show $R_0(t) = \beta(t)/\gamma(t)$ as a function of t.
