# Supplementary material for: Quantifying infectious disease epidemic risks: A practical approach for seasonal pathogens
Source: PLoS Comput Biol. 2025 Feb 19;21(2):e1012364. doi: 10.1371/journal.pcbi.1012364 (PMC11867399; doi:10.1371/journal.pcbi.1012364)
Supplement: S5 Fig — A. The TER (obtained by solving system of equations (11) from the main text numerically) when sustained transmission is only possible for a short period of the year (β0 = 1, β1 = 5 and γ = 4 . 9 month-1). Results are shown for both the time step used in the main text (Δt = 0 . 00033 months; blue line) and for a shorter time step (Δt = 0 . 00017 months; black dotted line). B. Analogous results to panel A, but with β0 = 3. C. Analogous results to panel A, but with β0 = 5. D. Analogous results to panel A, but with β0 = 7. E. Analogous results to panel A, but with β0 = 9. F. Analogous results to panel A, but with β0 = 11. In all panels, a threshold of M = 100 cumulative infections was used when computing the TER and the overall population size was assumed to be individuals. Insets show as a function of . (PDF) [file pcbi.1012364.s006.pdf]

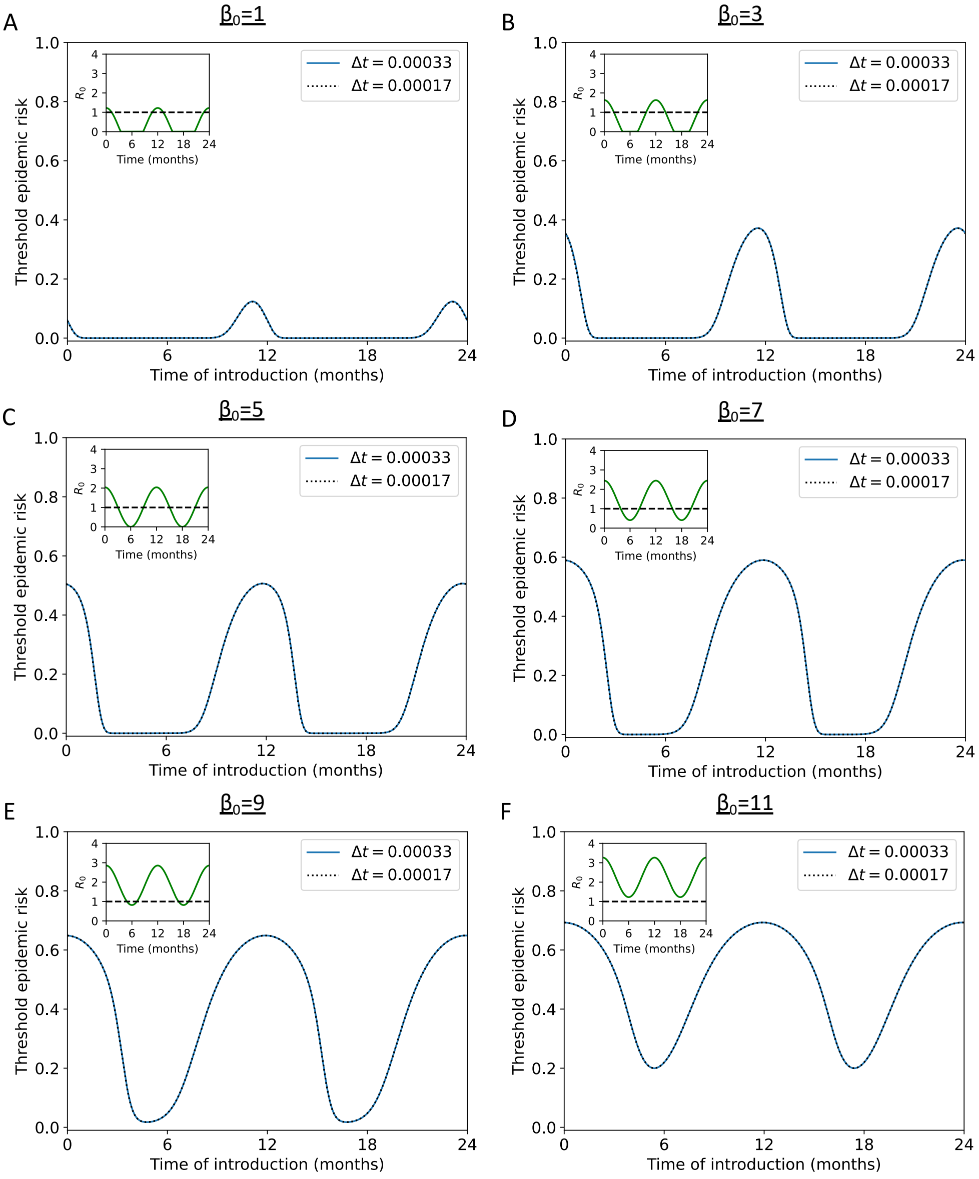

S5 Fig. Comparison between numerically computed values of the TER for two different values of the time step, Δt, for the stochastic SIR model with seasonal transmission. A. The TER (obtained by solving system of equations (11) from the main text numerically) when sustained transmission is only possible for a short period of the year ($\beta_0 = 1$, $\beta_1 = 5$ and $\gamma = 4.9$ month^{-1}). Results are shown for both the time step used in the main text ($\Delta t = 0.00033$ months; blue line) and for a shorter time step ($\Delta t = 0.00017$ months; black dotted line). B. Analogous results to panel A, but with $\beta_0 = 3$. C. Analogous results to panel A, but with $\beta_0 = 5$. D. Analogous results to panel A, but with $\beta_0 = 7$. E. Analogous results to panel A, but with $\beta_0 = 9$. F. Analogous results to panel A, but with $\beta_0 = 11$. In all panels, a threshold of $M = 100$ cumulative infections was used when computing the TER and the overall population size was assumed to be $N = 1{,}000$ individuals. Insets show $R_0(t) = \beta(t)/\gamma(t)$ as a function of t.
