# Supplementary material for: Quantifying infectious disease epidemic risks: A practical approach for seasonal pathogens
Source: PLoS Comput Biol. 2025 Feb 19;21(2):e1012364. doi: 10.1371/journal.pcbi.1012364 (PMC11867399; doi:10.1371/journal.pcbi.1012364)
Supplement: S6 Fig — A. The TER (obtained by solving system of equations (11) in the main text numerically) for a range of different values of the threshold number of infections, M. The blue shaded region shows the period of the year for which the TER exceeds z=0.4 when ,. B. The duration of the year for which the TER exceeds , shown as a function of M. C. Heatmap indicating the duration of the year for which the TER exceeds , shown for a range of values of M and z. In all panels, values of β0 = 4, β1 = 5 and γ = 4 . 9 month-1 are used. A time step of months was used when computing the TER. The overall population size was assumed to be N = 1 , 000 individuals. (PDF) [file pcbi.1012364.s007.pdf]

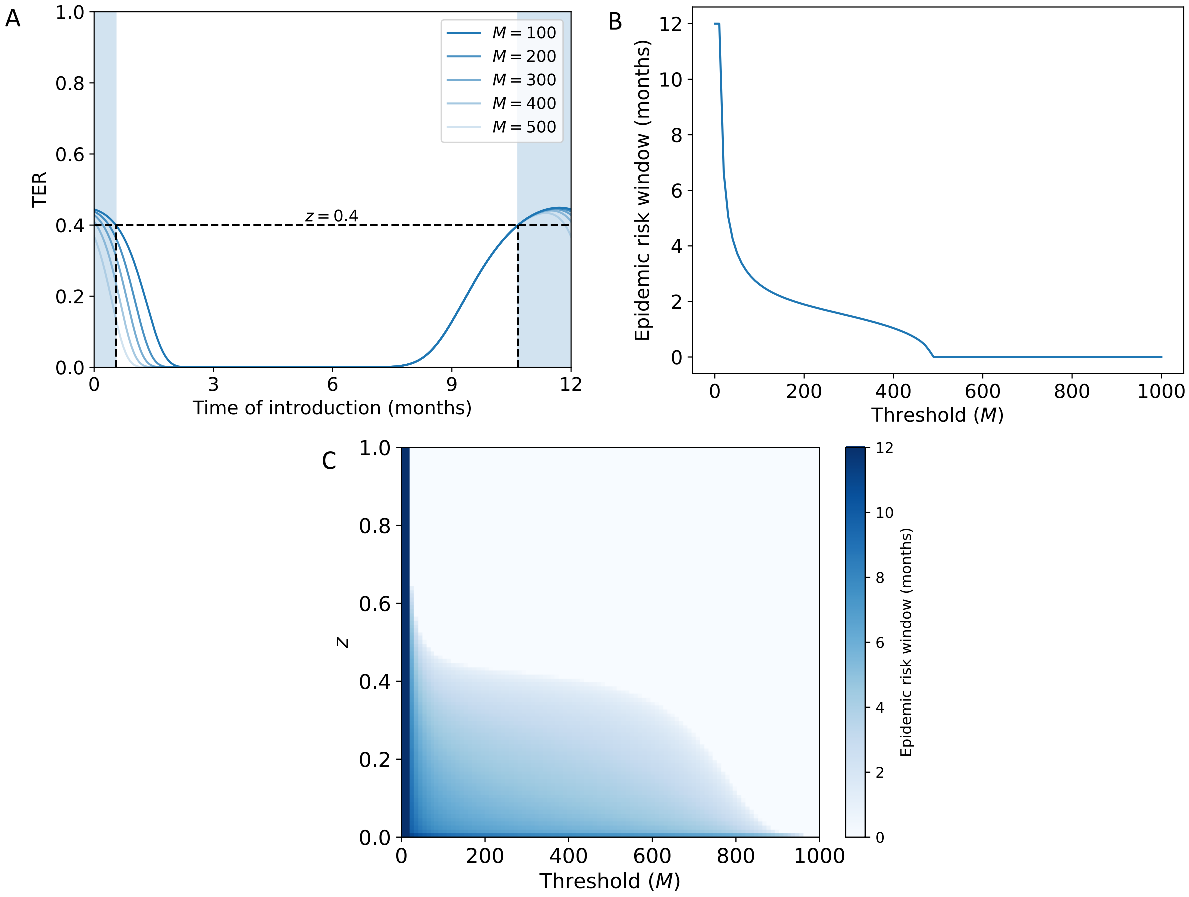

S6 Fig. Duration of the year for which the TER exceeds z in the stochastic SIR model with seasonal transmission, for a range of values of M and z. A. The TER (obtained by solving system of equations (11) in the main text numerically) for a range of different values of the threshold number of infections, M. The blue shaded region shows the period of the year for which the TER exceeds $z = 0.4$ when $M = 100$. B. The duration of the year for which the TER exceeds $z = 0.4$, shown as a function of M. C. Heatmap indicating the duration of the year for which the TER exceeds z, shown for a range of values of M and z. In all panels, values of $\beta_0 = 4$, $\beta_1 = 5$ and $\gamma = 4.9$ month^{-1} are used. A time step of $\Delta t = 0.00033$ months was used when computing the TER. The overall population size was assumed to be $N = 1{,}000$ individuals.
