# Supplementary material for: Quantifying infectious disease epidemic risks: A practical approach for seasonal pathogens
Source: PLoS Comput Biol. 2025 Feb 19;21(2):e1012364. doi: 10.1371/journal.pcbi.1012364 (PMC11867399; doi:10.1371/journal.pcbi.1012364)
Supplement: S7 Fig — A. The TER for 2014, shown for a range of values of the threshold number of cumulative infections, M. The TER computed using the approximation in equation (5) of the main text (as in Fig 5 of the main text; dashed lines) is compared to the TER computed using values of obtained directly from the numerical solution of the deterministic ecological model (system of equations (3) in the main text; solid lines). B. Analogous to panel A, but for 2015. In both panels, we ran 10,000 simulations of the stochastic model (using the simulation approach described in Section 2.1.2 of the main text) for each date of introduction considered. The host population size was assumed to be individuals (based on the population density in Feltre, this corresponds to an area of 80 Ha, and the numbers of adult female vectors (shown in S1C and S1D Fig) were scaled up from their per Ha values accordingly). (PDF) [file pcbi.1012364.s008.pdf]

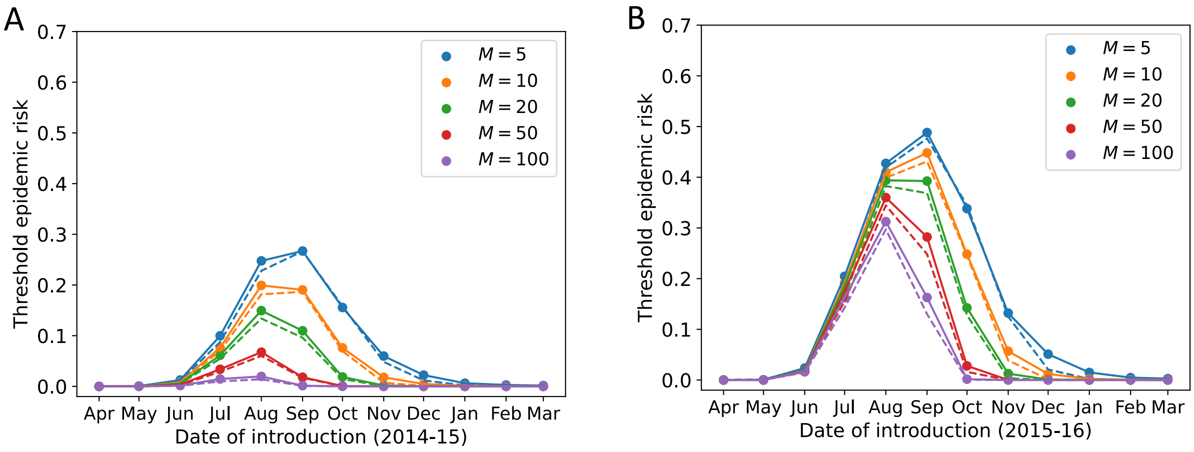

S7 Fig. Calculation of the TER for chikungunya in Feltre, Northern Italy, in 2014 and 2015, with and without the approximation in equation (5) of the main text. A. The TER for 2014, shown for a range of values of the threshold number of cumulative infections, M. The TER computed using the approximation in equation (5) of the main text (as in Fig 5 of the main text; dashed lines) is compared to the TER computed using values of $N_V(t)$ obtained directly from the numerical solution of the deterministic ecological model (system of equations (3) in the main text; solid lines). B. Analogous to panel A, but for 2015. In both panels, we ran 10,000 simulations of the stochastic model (using the simulation approach described in Section 2.1.2 of the main text) for each date of introduction considered. The host population size was assumed to be $N = 5{,}000$ individuals (based on the population density in Feltre [1], this corresponds to an area of 80 Ha, and the numbers of adult female vectors (shown in Fig S1.1C,D) were scaled up from their per Ha values accordingly).

References

1. Guzzetta G, Trentini F, Poletti P, Baldacchino FA, Montarsi F, Capelli G, et al. Effectiveness and economic assessment of routine larviciding for prevention of chikungunya and dengue in temperate urban settings in Europe. PLoS Negl Trop Dis. 2017;11. doi:10.1371/journal.pntd.0005918
